# Supplementary figures and images for: Reward network mechanism in anhedonia and depression
Source: PLoS One. 2025 Sep 18;20(9):e0332816. doi: 10.1371/journal.pone.0332816 (PMC12445566; doi:10.1371/journal.pone.0332816)

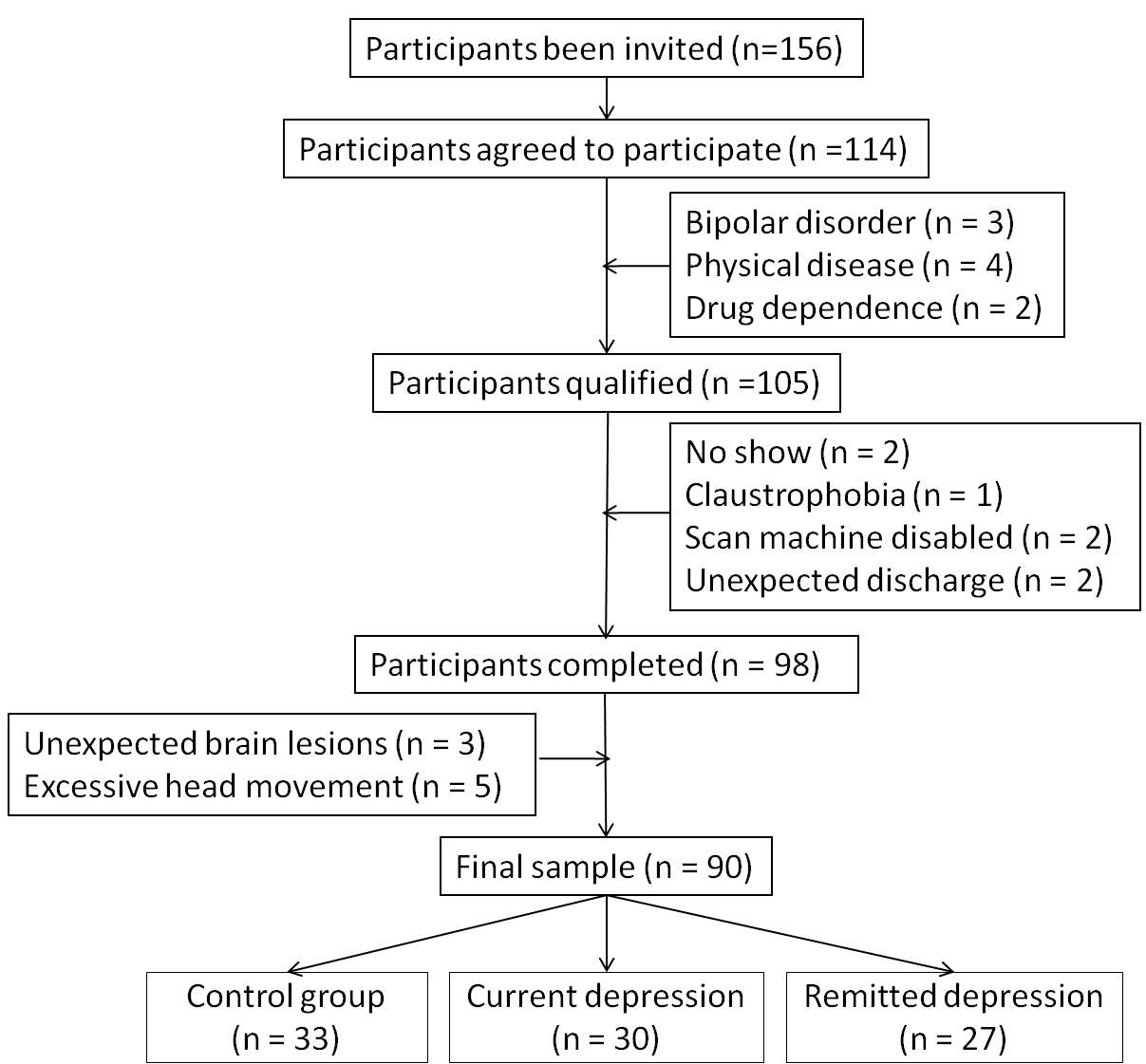

Supplement: S1 Fig — (TIF) [file pone.0332816.s001.tif]

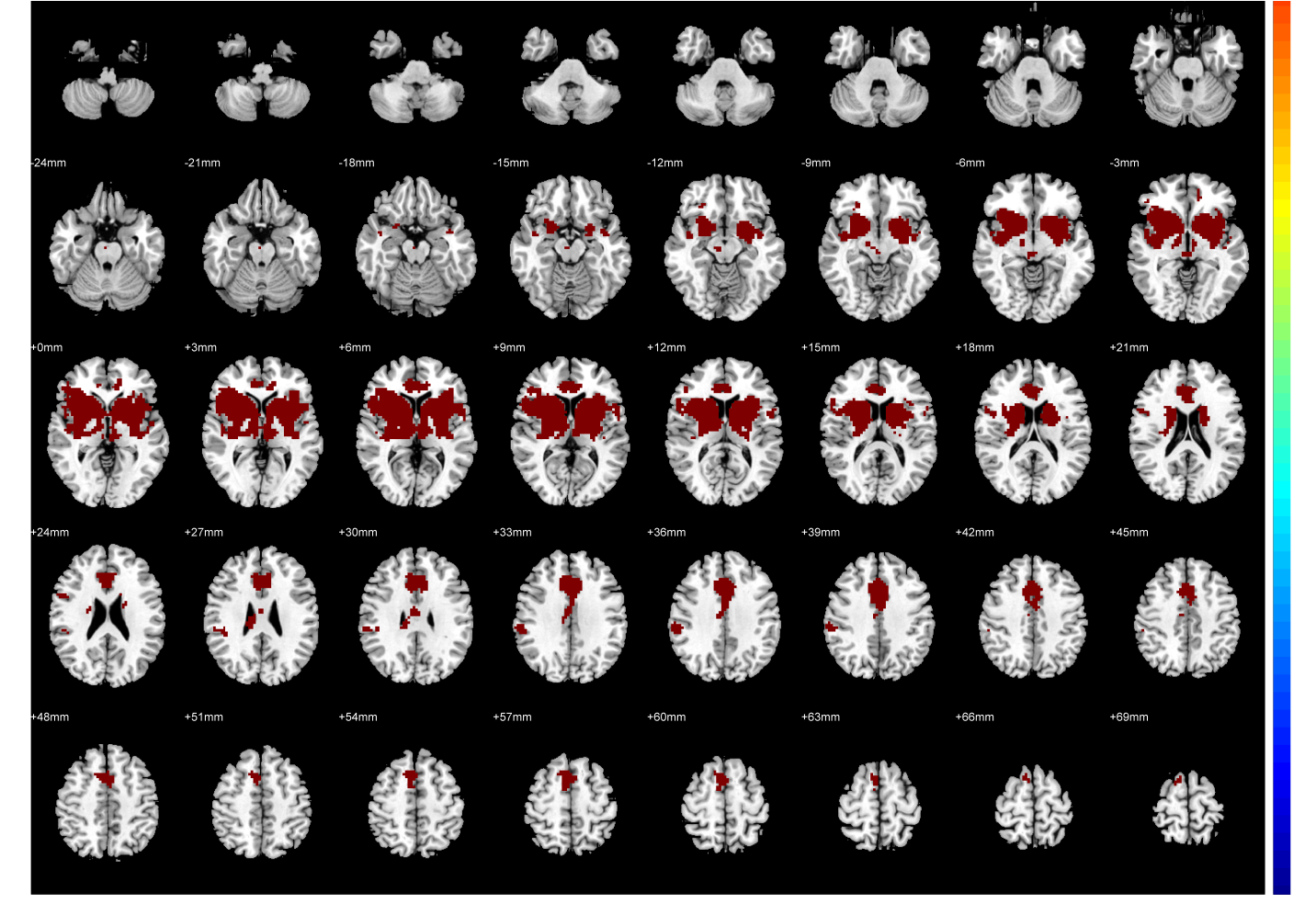

Supplement: S2 Fig — (TIF) [file pone.0332816.s002.tif]

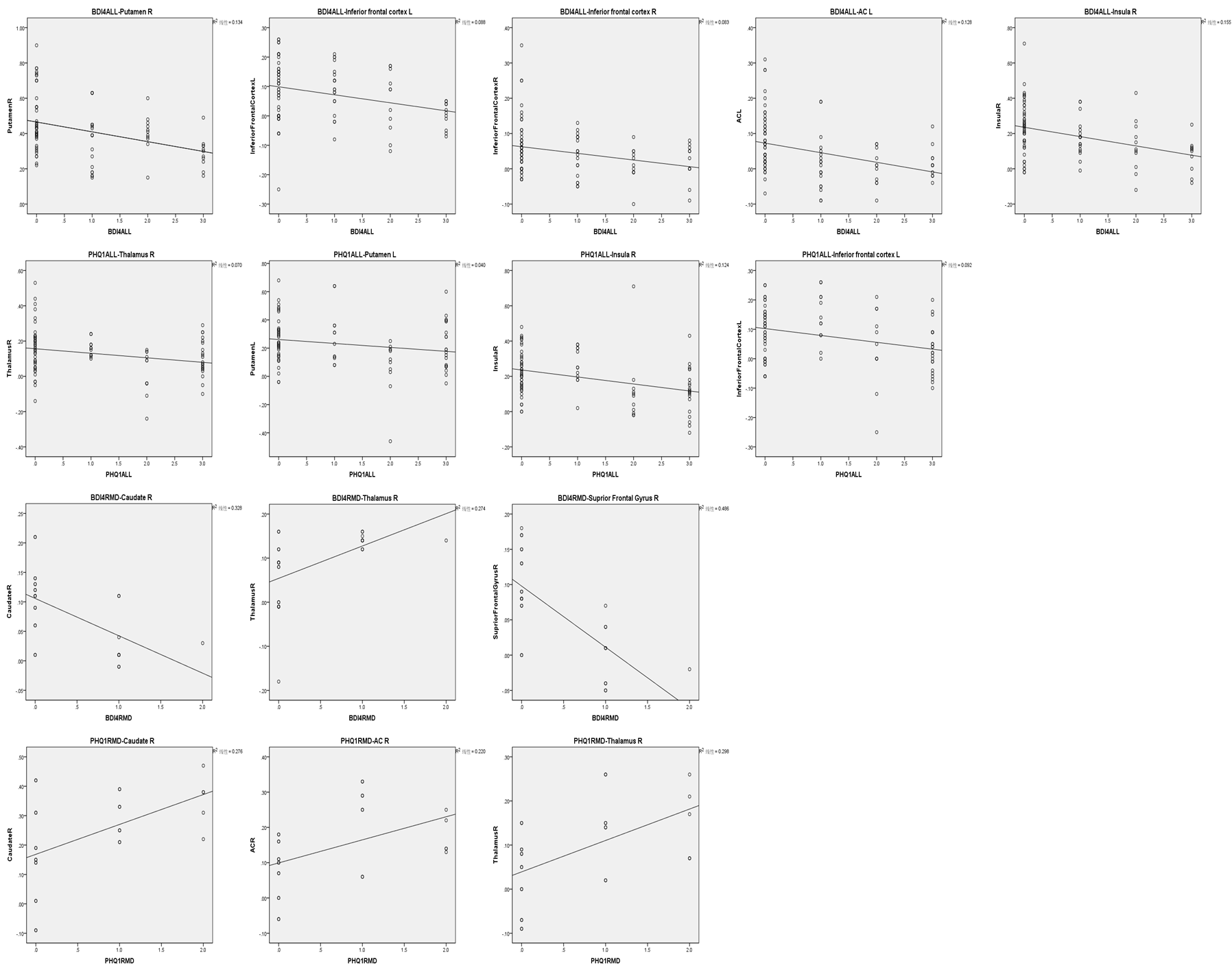

Supplement: S3 Fig — (TIF) [file pone.0332816.s003.tif]

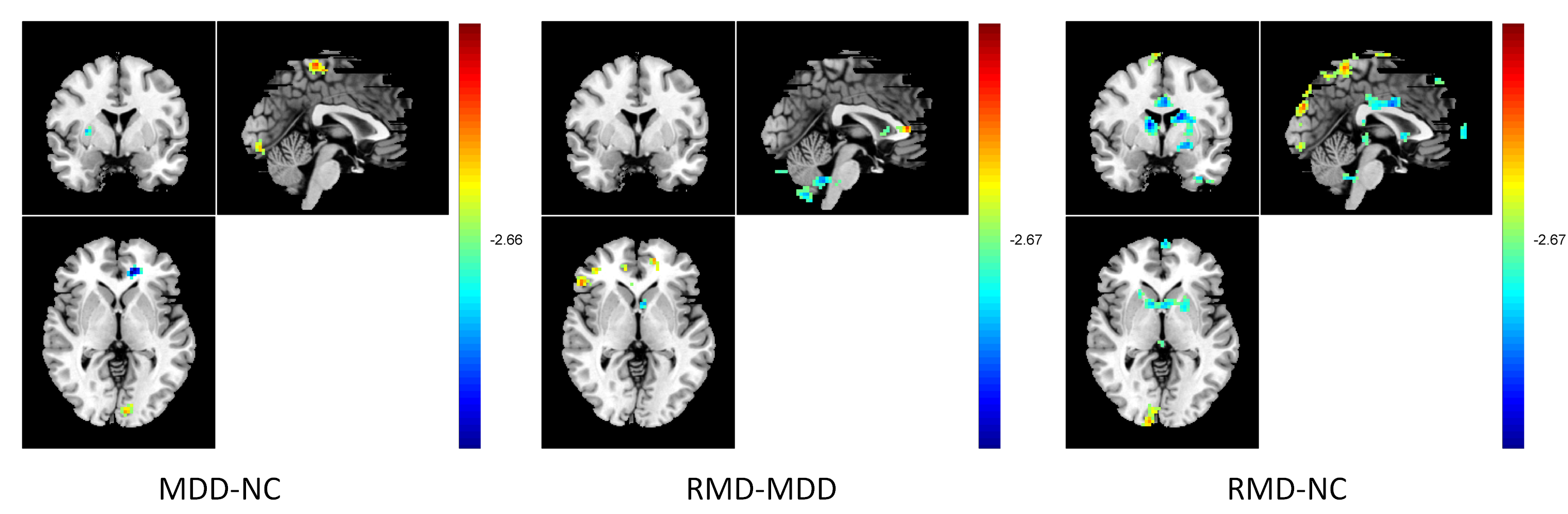

Supplement: S4 Fig — (TIF) [file pone.0332816.s004.tif]

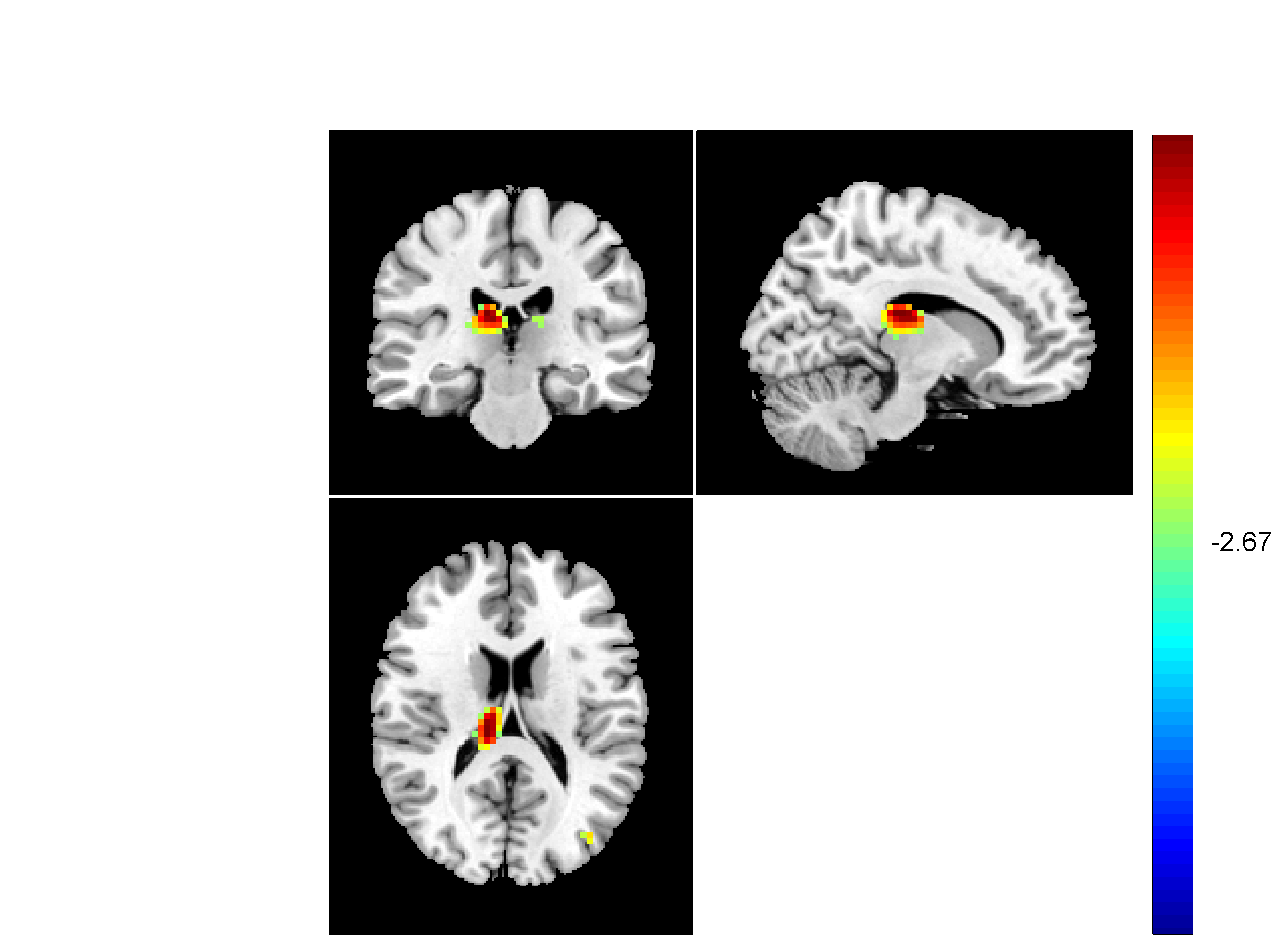

Supplement: S5 Fig — (TIFF) [file pone.0332816.s005.tiff]
