# Supplementary material for: Reward network mechanism in anhedonia and depression
Source: PLoS One. 2025 Sep 18;20(9):e0332816. doi: 10.1371/journal.pone.0332816 (PMC12445566; doi:10.1371/journal.pone.0332816)
Supplement: Attachment — Submitted filename: Revision interpretation for RN R1.docx [file pone.0332816.s008.docx]

**Dear Ph.D. Rosemary Bassey,**

Thank you for giving us an opportunity to revise the manuscript. Please find enclosed revised version of our manuscript entitled “Reward network mechanism in anhedonia and depression” for your consideration.

Many thanks for your constructive and detailed comments on the manuscript, which substantially improves the paper. In this revision version, we have addressed all of comments made by the reviewers (see below). With these changes, we hope that the manuscript will be of more relevant to the readership of your journal.

This manuscript is not in consideration elsewhere and the author reports no conflicts of interest. Moreover, the co-author has approved the revised version making significant contributions to the writing and conceptualization of the manuscript.

If I can be of any further assistance, please do not hesitate to contact me at my email address (daiqin101@hotmail.com). Thank you for your consideration.

Yours sincerely,

**Dai Qin**

**COMMENTS FOR THE AUTHOR:**

**Journal Requirements:**

When submitting your revision, we need you to address these additional requirements.

1. Please ensure that your manuscript meets PLOS ONE's style requirements, including those for file naming. The PLOS ONE style templates can be found at

https://journals.plos.org/plosone/s/file?id=wjVg/PLOSOne_formatting_sample_main_body.pdf and

[https://journals.plos.org/plosone/s/file?id=ba62/PLOSOne_formatting_sample_title_authors_affiliations.pdf](https://track.editorialmanager.com/CL0/https:%2F%2Fjournals.plos.org%2Fplosone%2Fs%2Ffile%3Fid=ba62%2FPLOSOne_formatting_sample_title_authors_affiliations.pdf/1/010f0197f054f7c6-687b2eee-68be-4100-bfa4-cff9193d1d4a-000000/Lzqg2OdDvuc2Smj4v5MGZYhSWL1kg4zzYfHOCyowFhg=219)

**Author’s responding:** Thank you for the detailed suggestion and revised accordingly.

2. Please describe in your methods section how capacity to provide consent was determined for the participants in this study. Please also state whether your ethics committee or IRB approved this consent procedure. If you did not assess capacity to consent please briefly outline why this was not necessary in this case.

**Author’s responding:** Thank you for the important suggestion and revised accordingly. The details about how capacity to provide consent in participants were added in **Participants (Line 80-82)** “…*The exclusion criteria were as follows: current or history of any psychopathology and/or drug and alcohol abuse infection, drug dependence, allergies within the last half month, physical trauma, severe physical disease, learning or cognitive disability*…” and **Procedure** **(Line 98-101,102-103)** “…*The study protocol including participants’ capacity to provide consent in accordance with the Declaration of Helsinki was approved by the Ethics Committee of Human Research of the Army Medical University (2020-036-02) and Chinese Clinical Trial registry center (ChiCTR2100044258)…The potential subjects (patients and normal controls) were screened for recruitment and their capacity to provide consent by trained personnel*…”

3. In the online submission form, you indicated that The data that support the findings of this study are available from the corresponding author upon reasonable request.].

All PLOS journals now require all data underlying the findings described in their manuscript to be freely available to other researchers, either 1. In a public repository, 2. Within the manuscript itself, or 3. Uploaded as supplementary information.

This policy applies to all data except where public deposition would breach compliance with the protocol approved by your research ethics board. If your data cannot be made publicly available for ethical or legal reasons (e.g., public availability would compromise patient privacy), please explain your reasons on resubmission and your exemption request will be escalated for approval.

**Author’s responding:** Thank you for the important suggestion and revised accordingly. We added the information in **Data Availability Statement “***The behavioral data that support the findings of this study were included in Supplementary materials. Due to reframing from military funding (2021HL003 and 2023yjsB02), the neural data could not be placed publicly and was available from the corresponding author upon reasonable request. To ensure the long term stability and availability of data, the neural data was packed and stored in three copies by a non-author institution (Military Psychology Committee institute, Chinese Society of Social Psychology) to ensure persistent or long-term data storage and availability, which was available from the official email address of this committee (*[*jsxlxzwh@163.com*](mailto:jsxlxzwh@163.com)*).*” and **Cover letter**.

4. Thank you for stating the following financial disclosure: [Doctor Dai claimed that this work was supported by the key project of natural science foundation of Chongqing (cstc2020jcyj-zdxmX0009), the Key project and innovation project of People's Liberation Army of China (2021HL003).].

Please state what role the funders took in the study.  If the funders had no role, please state: ""The funders had no role in study design, data collection and analysis, decision to publish, or preparation of the manuscript.""

If this statement is not correct you must amend it as needed.

Please include this amended Role of Funder statement in your cover letter; we will change the online submission form on your behalf.

**Author’s responding:** Thank you for the important suggestion and revised accordingly. See details in **Funding Declaration “***Doctor Dai claimed that this work was supported by Social Science Foundation of Chongqing (2023NDYB94), the Key project and innovation project of People's Liberation Army of China (2021HL003), and the education project of Army medical university (2023yjsB02). The funders had no role in study design, data collection and analysis, decision to publish, or preparation of the manuscript, only reframed the sharing of original neural data.*” and **Cover letter**.

5. Thank you for stating the following in the Competing Interests section: [Authors claimed receiving research funding from key project of nature science foundation of Chongqing (cstc2020jcyj-zdxmX0009), the Key project and innovation project of People's Liberation Army of China (2021HL003).Other authors report no biomedical financial interests or potential conflicts of interest.

All authors state that the content has not been published or submitted for publication elsewhere.].

Please confirm that this does not alter your adherence to all PLOS ONE policies on sharing data and materials, by including the following statement: ""This does not alter our adherence to  PLOS ONE policies on sharing data and materials.” (as detailed online in our guide for authors http://journals.plos.org/plosone/s/competing-interests).  If there are restrictions on sharing of data and/or materials, please state these. Please note that we cannot proceed with consideration of your article until this information has been declared.

Please include your updated Competing Interests statement in your cover letter; we will change the online submission form on your behalf.

**Author’s responding:** Thank you for the important suggestion and revised accordingly. See details in **Declaration of interest statement “***Authors claimed receiving research funding from Social Science Foundation of Chongqing (2023NDYB94), the Key project and innovation project of People's Liberation Army of China (2021HL003), and the education project of Army medical university (2023yjsB02). Other authors report no biomedical financial interests or potential conflicts of interest. This does not alter our adherence to PLOS ONE policies on sharing behavioral data, only limits on original neural data sharing from military funding (2021HL003 and 2023yjsB02). All authors state that the content has not been published or submitted for publication elsewhere.*” and **Cover letter**.

6. Thank you for stating the following in the Acknowledgments Section of your manuscript: [Authorsclaimed that this study was supported by key project of nature science foundation of Chongqing (cstc2020jcyj-zdxmX0009), the Key project and innovation project of People's Liberation Army of China (2021HL003). The authors thank clinical doctors who devote to patients recruitment, including Dr. Dr. L. D. Hu from Geleshan Psychiatry Hospital, Dr. X. Y. Zhou from Psychiatry center of Chongqing, Dr.Y.He from 324 Hospital of Chong Qing. We appreciate the endeavor of all graduate students who participated this study as research assistants. We also thank all subjects who took part in this study, especially the clinical patients.]

We note that you have provided funding information that is not currently declared in your Funding Statement. However, funding information should not appear in the Acknowledgments section or other areas of your manuscript. We will only publish funding information present in the Funding Statement section of the online submission form.

Please remove any funding-related text from the manuscript and let us know how you would like to update your Funding Statement. Currently, your Funding Statement reads as follows: [Doctor Dai claimed that this work was supported by the key project of natural science foundation of Chongqing (cstc2020jcyj-zdxmX0009), the Key project and innovation project of People's Liberation Army of China (2021HL003).]. 
Please include your amended statements within your cover letter; we will change the online submission form on your behalf.

**Author’s responding:** Thank you for the important suggestion and revised accordingly. See details in **Acknowledgement “***The authors thank clinical doctors who devote to patients recruitment, including Dr. Dr. L. D. Hu from Geleshan Psychiatry Hospital, Dr. X. Y. Zhou from Psychiatry center of Chongqing, Dr.Y.He from 324 Hospital of Chong Qing. We appreciate the endeavor of all graduate students who participated this study as research assistants. We also thank all subjects who took part in this study, especially the clinical patients.*” and **Cover letter**.

7. Your ethics statement should only appear in the Methods section of your manuscript. If your ethics statement is written in any section besides the Methods, please move it to the Methods section and delete it from any other section. Please ensure that your ethics statement is included in your manuscript, as the ethics statement entered into the online submission form will not be published alongside your manuscript.

**Author’s responding:** Thank you for the valuable suggestion and revised accordingly. Ethic statement were moved to **Procedures (Line 96-101):** “*All procedures performed in studies involving human participants were in accordance with the ethical standards of the institutional and/or national research committee and with the 1964 Helsinki Declaration and its later amendments or comparable ethical standards. The study protocol including participants’ capacity to provide consent in accordance with the Declaration of Helsinki was approved by the Ethics Committee of Human Research of the Army Medical University (2020-036-02) and Chinese Clinical Trial registry center (ChiCTR2100044258)*…”

8. We notice that your supplementary figures are uploaded with the file type 'Figure'. Please amend the file type to 'Supporting Information'. Please ensure that each Supporting Information file has a legend listed in the manuscript after the references list.

**Author’s responding:** Thank you for the important suggestion and revised accordingly. All supplementary Figures were uploaded as 'Supporting Information'.

9. We notice that your supplementary figures are included in the manuscript file. Please remove them and upload them with the file type 'Supporting Information'. Please ensure that each Supporting Information file has a legend listed in the manuscript after the references list.

**Author’s responding:** Thank you for the important suggestion and revised accordingly. All supplementary Figures had a legend listed in the manuscript after the references list and were uploaded as 'Supporting Information'.

10. Please review your reference list to ensure that it is complete and correct. If you have cited papers that have been retracted, please include the rationale for doing so in the manuscript text, or remove these references and replace them with relevant current references. Any changes to the reference list should be mentioned in the rebuttal letter that accompanies your revised manuscript. If you need to cite a retracted article, indicate the article’s retracted status in the References list and also include a citation and full reference for the retraction notice.
**Author’s responding:** Thank you for the important suggestion and revised accordingly. We checked all references and made minor revisions in volume and pages in some of **references (11, 12, 45)**, and confirmed that the references were complete and correct in the current version.

**Reviewer #1:**

The manuscript by Sun D.X. and co-authors analyses the clinical data on anhedonia and depression from the side of reward network mechanism.

The manuscript is well-prepared, gives enough data to confirm the author's statements and claims.
With this in mind, presented manuscript is a good work that may be published in PLOS One journal.

**Author’s responding:** Many thanks for your constructive and detailed comments on the manuscript, which substantially improves the paper.

Per contrary, it has several important points I would like to address to the authors and I expect they will correct them:

1. Please discuss the recent data on brain structures (that you study in your work) interactions in regulation of anhedonia and depression. I suppose there are a lot of new experimental articles that show how these brain regions interact with each other in controlling behavior employing cell and tissue histology and physiology methods.

**Author’s responding:** Thank you for the valuable suggestion and revised accordingly. We described latest reporting about neural mechanism in regulation of anhedonia and depression in **1st and 2nd paragraph of Introduction (Line 12-14, 29-32)**: “…*Blunted left ventral striatum response to reward was associated with a lifetime depression diagnosis [**8**]. Recent review indicated that compared with healthy controls, patients with MDD exhibited common activity decreases in the right striatum (putamen, caudate) and subgenual ACC [**9**]…. Change in anhedonia scores negatively correlated with rsFC after antidepressant treatment [**17**]. A previous study further reported that anhedonia symptom and depressive mood were associated with similar striatal circuits in MDD patients with money-reward task [**16**]. Indeed, RN was indicated associated with both anhedonia and depression [**18**]*…”

2. The references list is very old. Two articles published on 2020 and 2021 are the newest ones in this manuscript. The authors should update the references list by adding the newer publications.

**Author’s responding:** Thank you for the important suggestion and revised accordingly. We added 14 recent literatures in this version (literature 6, 8, 9, 17, 18, 19, 21-22, 24-26, 35, 46, 47). See **references** for details.

3. In addition to the study limitations, the authors should also include the perspectives subsection, where they should write in details on how potentially this work and the data may be used in further research.

**Author’s responding:** Thank you for the inspiring suggestion and revised accordingly. A separate paragraph about the perspective of current findings were added **after Limitation and before Conclusion (Line 293-302)**: “*Notably, this study included patients with current and remitted depression and confirmed that MDD and RMD patients possess different RN pattern, as well as depressive episode and anhedonia. The findings help to suggest candidate cortex in the treatment of depression. Specifically, caudate involves more in remitted depression, which might be an indicator of remitted stage, and be a potential target in future antidepressant treatment such as deep brain stimulation or medication. Putamen indicates only anhedonia but not depressive episode, which might be used to discriminate anhedonia and depressive episode. Insula and inferior frontal involve in both depressive episode and anhedonia, which might be cortex markers of both situations. Notably, AC and thalamus are trait-like markers of both current and remitted depression, which might involve in different stages of depressive progress, and reflected trait-like neural changes in depression. Future neural experiments including both MDD and RMD are warranted to confirm these findings.*”

4. Sometimes the English usage appears strange, please check the spelling.

**Author’s responding:** Thank you for the important suggestion and revised accordingly. We read and revised the language throughout the manuscript, and further invited an English-speaking scholar to read and revise the grammatically issues and typos throughout the manuscript and Tables.

**Reviewer #2:**

This study is an interesting research topic that has demonstrated the different reward network involvement of anhedonia and depression. However, in order to increase readers' understanding, it seems necessary to revise the following parts.

**Author’s responding:** Many thanks for your constructive and detailed comments on the manuscript, which substantially improves the paper.

1. Throughout the manuscript, certain words (aka. correlate) are repeated too much, and the fact that it is unnecessarily the first study is used repeatedly for discussion. English correction is needed throughout the paper.

**Author’s responding:** Thank you for the valuable suggestion and revised accordingly. Two of the descriptions of “first study” in Discussions were deleted, see **1st and 2nd paragraph of Discussions (Line 216 and 222) f**or details: “*The current study found that RMD patients possess different RN pattern of anhedonia…This study reports and compares anhedonia symptoms of current and remitted depression in one single study, which enriches the understanding about the anhedonia during the process of depression…*”. We further read and revised the language throughout the manuscript, and further invited an English-speaking scholar to read and revise the grammatically issues and typos throughout the manuscript and Tables.

2. With this study alone, it seems unreasonable for the reward network to evaluate which of the state or trace markers will be suitable. Nevertheless, if the authors describe it as suitable as a trace marker, the evidence for this should be described.
**Author’s responding:** Thank you for the valuable suggestion and revised accordingly. We added the rationale why including RMD may indicate a trait-like marker in the **3rd paragraph of Introduction (Line 43-45)**: “…*Comparison between current and remitted depression may suggest trait- or state- marker of depression, i.e., commonality between them suggests a trait-like marker while difference between them suggests a state-like marker [**24-26**]*….”

3. In the introduction, authors argued that anhedonia is a core symptom of depression, and MDD and anhedonia may have different natural mechanisms. Could you explain this issue more detail in evidence?
**Author’s responding:** Thank you for the valuable suggestion and revised accordingly. We added more deduction about why MDD and anhedonia may have different natural mechanisms in the **2nd paragraph of Introduction (Line 33-42)**: “…*However, anhedonia is a core symptom of depressive episode, anhedonia itself can not represent a depressive episode [**19**]. Specifically, to be diagnosed with MDD, one must be in depressed mood or anhedonia which causes social/occupational impairment [**20**], i.e., anhedonia is only one symptom of MDD which might be exempted from experiencing by some patients with MDD [**19**]. If some patients experience anhedonia while others not, it thus could be deduced that the neural mechanism underling MDD may be differed but alike with anhedonia (****Hypothesis 1****), since that MDD was formed by broader symptoms such as sadness or insomnia which were very different from anhedonia [**21**,* *22**]. However, further evidence is needed to confirm whether anhedonia symptom and depressive episode share similar but subtly-different resting-state reward network mechanism, clarifying this issue might be important to suggest candidate cortex for clinical depression therapy, especially for anhedonia-type depression.*”

4. pp. 42-44. When describing the conflicting results of previous studies on decreased depression, hyperactivation was described before without mentioning a specific brain region, and hypoactivation was described later in putamen and caudate. The authors should specify the specific brain region that is hyperactivated.
**Author’s responding:** Thank you for the valuable suggestion and revised accordingly. The specific hyperactivated brain regions were specified. See details in the **3rd paragraph of Introduction (Line 50-53)**: “…*A limited number of researches reported that remitted depression has a different RN activation during a reward process (hyperactivation while anticipating a reward in bilateral anterior cingulate gyrus and right midfrontal gyrus and hypoactivation during reward outcomes in orbital frontal cortex, right frontal pole, left insular cortex, and left thalamus) [**28**]*….”

5. In the last part of the introduction, the authors established two hypotheses, talking about the purpose of the study. The authors should further describe the scientific basis for this hypothesis.
**Author’s responding:** Thank you for the valuable suggestion and revised accordingly. The scientific basis for the two hypotheses was added in the Introduction. See details in the **2nd (Line 33-42)**“…*However, anhedonia is a core symptom of depressive episode, anhedonia itself can not represent a depressive episode [**19**]. Specifically, to be diagnosed with MDD, one must be in depressed mood or anhedonia which causes social/occupational impairment [**20**], i.e., anhedonia is only one symptom of MDD which might be exempted from experiencing by some patients with MDD [**19**]. If some patients experience anhedonia while others not, it thus could be deduced that the neural mechanism underling MDD may be differed but alike with anhedonia (****Hypothesis 1****), since that MDD was formed by broader symptoms such as sadness or insomnia which were very different from anhedonia [**21**,* *22**]. However, further evidence is needed to confirm whether anhedonia symptom and depressive episode share similar but subtly-different resting-state reward network mechanism, clarifying this issue might be important to suggest candidate cortex for clinical depression therapy, especially for anhedonia-type depression.*” and **3rd paragraph (Line 43-45, 53-59) of Introduction**: “*Importantly, depression is a highly recurrent mood disorder [**23**]. Comparison between current and remitted depression may suggest trait- or state- marker of depression, i.e., commonality between them suggests a trait-like marker while difference between them suggests a state-like marker [**24-26**]…It is well known that the current and remitted depression differ in symptoms [**31**] and neural foundation [**25**,* *26**], it could be assumed that they differ significantly in resting-state RN mechanism (****Hypothesis 2****). Therefore, this study included patients with remitted depression (RMD) to confirm the role of RN mechanism during the remission of depression, and further compare with that of MDD to confirm whether RN mechanism is a state marker of depressive episode or a trait-like mechanism related to the occurrence and recurrence of depression.*”

6. In Figures 1-5, specific positions with the annotation (A,B,C) should be established and specify each figure location in the manuscript.

**Author’s responding:** Thank you for the important suggestion and revised accordingly. We put specific positions with the annotation (A,B,C) in Figures and specified each figure location in the manuscript. See **Figures and Results** “**Correlation between functional connectivity and anhedonia (Table 3)**

To observe the relationship between neural function connectivity and depressive anhedonia, correlations between the value of resting-state RN connectivity and the anhedonia scores in all participants and sub-groups were conducted using REST 1.8. In total sample, a negative correlation was found between the severity of anhedonia (4^th^ item of BDI-II, Fig1A) and striatum functional connectivity in the right putamen (r = -0.39, k = 14, p < 0.05), left inferior frontal cortex (r = -0.31, k = 21, p < 0.05), right inferior frontal cortex (r = -0.38, k = 47, p < 0.05), left AC (r = -0.41, k = 57, p < 0.05), and right insula (r = -0.43, k = 43, p < 0.01); in addition, a negative correlation was found between the frequency of anhedonia (1^st^ item of PHQ, Fig 1B) and striatum functional connectivity in the right insula (r = -0.42, k = 24, p < 0.05), left inferior frontal cortex (r = -0.33, k = 21, p < 0.05), left putamen (r = -0.29, k = 24, p < 0.05), and right thalamus (r = -0.34, k = 27, p < 0.05). In the RMD group, a positive correlation was found between the severity of anhedonia (Fig 2A) and striatum functional connectivity in the right thalamus (r = 0.78, k = 50, p < 0.05); a negative correlation was found between the severity of anhedonia and striatum functional connectivity in the right caudate (r = -0.65, k = 12, p < 0.05) and right superior frontal gyrus (r = -0.81, k = 46, p < 0.01); and a positive correlation was found between the frequency of anhedonia (Fig 2B) and striatum functional connectivity in the right caudate (r = 0.77, k = 38, p < 0.01), right AC (r = 0.79, k = 50, p < 0.01), and right thalamus (r = 0.77, k =23, p < 0.01). No significant correlation between anhedonia and striatum connectivity was found in the NC and MDD groups. See Figs1–2, Table 3, and S3 Fig (scatter plots) for details.

**Functional connectivity (Table 4)**

One-way ANOVA (analyzed using REST 1.8) on the functional connectivity between the striatum seed and RN indicated that the three groups differed in their connectivity between the striatum seed and the left caudate (k = 21, p < 0.01), the left anterior (k = 43, p < 0.05), and the middle cingulate gyrus (k = 24, p < 0.05), S4 Fig. Further two-sample t-tests (AlphaSim correction, corrected p-value < 0.01) indicated that compared with the normal controls, the patients with current depression exhibited weaker connectivity between the striatum seed and the right insula (k = 27, p < 0.01), the right inferior frontal cortex (k = 130, p < 0.05), and the left anterior cingulated gyrus (AC) (k = 119, p < 0.05) (**Fig 3**). In contrast, compared with the MDD group, individuals with remitted depression showed stronger connectivity between the striatum seed and the right inferior frontal cortex (k = 55, p < 0.05), the right insula (k = 20, p < 0.05), and the right middle frontal gyrus (k = 43, p < 0.05) and weaker connectivity between the striatum seed and the left and right caudate (k = 27, k = 26, p < 0.05) (**Fig 4**). They also showed weaker connectivity between the striatum seed and the left caudate (k = 117, p < 0.01) and the cingulated gyrus (k = 76, p < 0.01) compared with the normal controls (**Fig 5**). ”for details.

**Reviewer #3:**

This is an observational study with a cross-sectional design that employed resting-state functional magnetic resonance imaging (rs-fMRI) to investigate patterns of functional brain connectivity. The primary objective was to compare connectivity differences across three groups: individuals with remitted depression, individuals currently experiencing a depressive episode, and individuals with no history of psychiatric disorders. The methodology was clearly described, utilizing standardized and validated instruments appropriate for the target population and suitable for the collection and analysis of neurofunctional data.
**Author’s responding:** Many thanks for your constructive and detailed comments on the manuscript, which encourage us a lot.

**Thank you for your valuable comments and giving us an opportunity to revise our manuscript. We hope that we have addressed the comments satisfactorily in this revision. If there are other errors or further requests, please contact us by e-mail (**[**daiqin101@hotmail.com**](mailto:daiqin101@hotmail.com)**).**

**Finally, I would like to thank you for your excellent suggestions which are so helpful to enhancing our study.**
